# Acute Lower Limb Ischemia in COVID-19 Patient with Delayed Presentation

**DOI:** 10.1155/2021/3333057

**Published:** 2021-12-08

**Authors:** Sangam Shah, Rukesh Yadav, Rajan Chamlagain, Yagya Raj Adhikari, Sanjit Kumar Sah, Bipin Kandel, Subash Basnet, Sutap Yadav

**Affiliations:** ^1^Maharajgunj Medical Campus, Institute of Medicine, Tribhuvan University, Maharajgunj 44600, Nepal; ^2^Tribhuvan University Teaching Hospital, Maharajgunj 44600, Nepal; ^3^Department of Cardiology, Institute of Medicine, Tribhuvan University, Maharajgunj 44600, Nepal; ^4^Department of Cardiology, Manmohan Cardiothoracic Vascular and Transplant Center, Maharajgunj 44600, Nepal

## Abstract

Thromboembolism is a common complication of SARS-CoV-2, which generally involves venous thromboembolism, although there have been reported cases of arterial thrombosis affecting cerebral, coronary, and visceral arteries, as well as arteries in the extremities. We discuss a case of a 45-year-old diabetic man with COVID-19 who developed late-onset acute lower limb ischemia.

## 1. Introduction

Coronavirus disease 19 (COVID-19) is a contagious disease caused by severe acute respiratory syndrome coronavirus-2 (SARS-CoV-2) that was first reported on December 2019 in Wuhan, China. As of July 2021, about 19 million cases have been reported across the world with approximately 4 million deaths. The presentation of COVID-19 ranges from asymptomatic to a rapidly progressive fatal disease, which may result from multiorgan failure, mainly involving the respiratory system. Severe acute respiratory syndrome coronavirus-2 causes coagulation problems that lead to thromboembolic consequences (SARS-CoV-2) [1]. High D-dimer, von Willebrand factor antigen and activity, and factor VIII activity are noted in patients with COVID-19 having coagulation disorders. Endothelial dysfunction, inflammation, cytokine release, hypercoagulability, and hypoxia all have been linked to thrombosis [2]. We discuss the case of a 45-year-old diabetic man who developed left lower leg ischemia eighteen days after the diagnosis of COVID pneumonia.

## 2. Case Presentation

A 45-year-old diabetic male presented to our center with chief complaints of bluish discoloration and cold left lower limb for five days. He also complained of left lower limb pain and subsequent difficulty in walking. He had COVID-19 that was confirmed from nasopharyngeal swab by reverse transcriptase polymerase chain reaction (RT-PCR) 18 days prior to admission to our center. He was managed conservatively with ceftriaxone, azithromycin, and dexamethasone. He consumed 4-6 cigarettes per day for 20 years but did not consume alcohol.

He was ill looking and not well oriented to time, place, and person with Glasgow Coma Scale (GCS) of 12/15. His axillary temperature was 97.9°F, blood pressure was 100/70 mm Hg, respiratory rate was 24 breaths/minute, pulse rate was 120 beats per minute, and oxygen saturation was 90% in the room air. On general physical examination, he had no pallor, icterus, clubbing, edema, and cyanosis. Dorsalis pedis artery and posterior tibial artery of the left lower limb were feeble on palpation.

**Figure 1:**
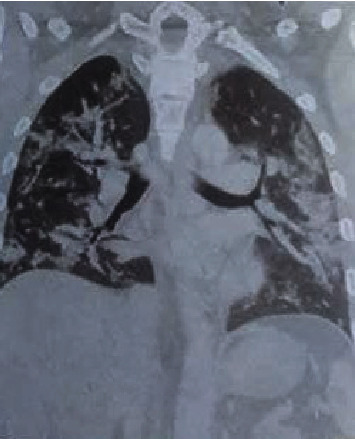
HRCT of chest showing multifocal consolidation with ground glass opacities, interlobular septal thickening, and fibrotic bands in the lungs.

**Figure 2:**
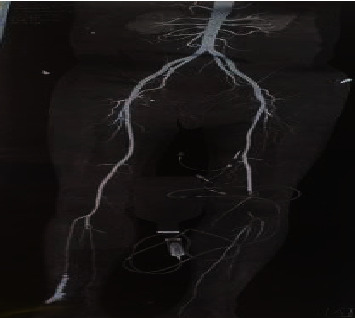
CT angiogram of abdominal aorta and bilateral lower limbs showing partially occluded thrombus in bilateral profunda femoris artery, completely occluding thrombus in left popliteal artery and tibioperoneal trunk.

Laboratory examination revealed hemoglobin 11.6 gm%, total leukocyte count 15800 cells/mm^3^, platelets 506000 cells/mm^3^, urea 7 mg/dl, creatinine 0.6 mg/dl, Na^+132^ mEq/l, and K^+4.1^ mEq/l at the time of presentation. His random blood sugar was 283 mg/dl, and activated partial thromboplastin time (APTT) was 110 seconds. Arterial blood gas (ABG) analysis revealed respiratory alkalosis and hypoxemia. High-resolution computed tomography (HRCT) of chest showed multifocal consolidation with ground glass opacities, interlobular septal thickening, and fibrotic bands in the lungs (Figure 1). CT angiogram of abdominal aorta and bilateral lower limbs showed partially occluded thrombus in bilateral profunda femoris artery, completely occluding thrombus in left popliteal artery and tibioperoneal trunk and partially extending to left anterior tibial artery with collateral reformation of left posterior tibial artery and peroneal artery (Figure 2).

Clinically, he had Rutherford class I acute limb ischemia, and hence, he was planned for anticoagulation with unfractionated heparin (UFH). Furthermore, the patient was managed with intravenous antibiotic (ceftriaxone), analgesic (paracetamol and tramadol), rosuvastatin, metformin, aspirin (75 mg OD), cilostazol (100 mg BD), and insulin. He was kept on UFH for 10 days during hospital stay. The patient showed improvement as pain subsided and patient could walk without difficulty again, and following this, he was discharged on warfarin for a month.

On a follow-up visit after one month, he had difficulty in walking along with pain and swelling in the left lower limb. Power was 4/5 in the left knee flexion and extension, ankle extension, and dorsiflexion. Sensation for touch and temperature was decreased in the left leg. Angiogram and Doppler study showed organized clot at the level of the popliteal artery with distal flow. He was diagnosed with occlusive disease of left popliteal artery. Following this, he was managed by distal embolectomy of left superficial femoral artery. He was discharged after five days on oral medications (rivaroxaban (20 mg OD), rosuvastatin (20 mg OD), and metformin (500 mg BD)). On a follow-up after 12 days of embolectomy, he had no complications and was able to walk without pain.

## 3. Discussion

Even with mild symptoms, COVID-19 patients might develop acute limb ischemia as seen in our case. Although acute respiratory distress syndrome (ARDS) is the most common consequence in patients with severe COVID-19, cardiovascular, thromboembolic, inflammatory, and neurologic complications are being reported more recently. Comorbidities such as cardiovascular disease, diabetes mellitus, obesity, smoking, cancer, chronic renal disease, chronic obstructive pulmonary disease, solid organ or hematopoietic stem cell transplantation, and advanced age are the risk factors for developing severe COVID-19 infection [3, 4]. Coagulation disorders that lead to thromboembolic events are linked to a poor prognosis. According to the study by Fournier et al. conducted in France, COVID patients with arterial thrombosis had a threefold greater mortality [5].

Embolization of a cardiac or aneurysmal thrombus or thrombophilic conditions such as antiphospholipid antibody syndrome, malignancy, or heparin-induced thrombocytopenia are the most common differential diagnoses in a patient with thrombotic abnormality. Given the huge clot size and the absence of clinical signs of endocarditis, arrhythmia, or congestive heart failure, a cardioembolic cause was ruled out in our patient. Similarly, his clinical presentation, laboratory testing, and chest imaging did not indicate the existence of a malignancy, and once COVID-19 was diagnosed, no further workup was done [6, 7]. Patients with comorbidities including hypertension, diabetes, chronic respiratory disease, renal disease, and obesity have a higher risk of adverse outcomes [8].

While the majority of patients with arterial thrombosis in the literatures had the aforementioned preexisting comorbidities, it is important to note that a minority of patients had no prior health problems or arterial disease [9]. Furthermore, the majority of documented cases of acute limb ischemia occurred in hospitalized patients to patients admitted in high dependency or intensive care units. The most common thrombotic event is venous thromboembolism, but there have been instances of arterial events such as stroke, myocardial infarction, and acute limb ischemia. Acute limb ischemia can occur with asymptomatic to moderately symptomatic patients who are hospitalized in the intensive care unit (ICU) due to severe COVID-19 infection.

Virchow triad explains the pathophysiology of arterial and venous thrombosis (hypercoagulability, stasis, and endothelial injury). Endothelial damage caused direct invasion of endothelial cells, and complement activation has been identified as another mechanism [10]. Endothelial damage can also be caused by acute-phase reactants and acute inflammatory response mediators. Blood stasis plays a role in critically ill patients with longer period of immobilization. COVID-19 patients have hyperviscosity, increased levels of factor VIII, fibrinogen, circulating thrombotic microparticles, and neutrophil extracellular traps. The suppression of complement activation prevents the thromboembolic events that also include limb ischemia and end-organ damage [11]. Bellosta et al. recently published a study that found a low revascularization success rate in a group of 20 patients [12]. Due to the low success rate of revascularization, conservative treatment with anticoagulation is indicated. Our treatment was consistent with the European Society of Vascular Surgery (ESVS) guidelines, which encourage conservative management in patients with class I acute limb ischemia [13]. However, our patient's symptoms did not improve; therefore, embolectomy was performed later.

## 4. Conclusion

Even with mild symptoms, COVID-19 patients might develop acute limb ischemia, thrombotic events like venous thromboembolism, thrombotic stroke, and acute myocardial infection. If the limb is not urgently threatened, it can be treated conservatively with UFH and with surgical embolectomy as a last resort if the symptoms do not improve. Anticoagulation alone may not be enough to relieve the symptoms of acute limb ischemia, and the ischemia may progress to the point where the limb cannot be saved. As observed in our patient, thrombotic events can occur even late in the course after COVID-19 detection.

## Data Availability

All the required information is available in the manuscript itself.
